# Evaluation of the mechanism of action of paracetamol, drotaverine, and peppermint oil and their effects in combination with hyoscine butylbromide on colonic motility: human *ex-vivo* study

**DOI:** 10.3389/fphar.2024.1384070

**Published:** 2024-07-10

**Authors:** Sara Traserra, Claudia Barber, Luis Gerardo Alcalá-González, Stefania Landolfi, Robert Lange, Carolina Malagelada, Maura Corsetti, Marcel Jimenez

**Affiliations:** ^1^ Department of Cell Biology, Physiology and Immunology, Universitat Autònoma de Barcelona, Barcelona, Spain; ^2^ Digestive System Research Unit, Vall d'Hebron University Hospital, Barcelona, Spain; ^3^ Department of Pathology, Vall d'Hebron University Hospital, Barcelona, Spain; ^4^ Sanofi, Frankfurt am Main, Frankfurt, Germany; ^5^ Centro de Investigación Biomédica en Red de Enfermedades Hepáticas y Digestivas (CIBEREHD), Barcelona, Spain; ^6^ NIHR Nottingham Biomedical Research Centre (BRC), Nottingham University Hospitals NHS Trust and the University of Nottingham, Nottingham, United Kingdom; ^7^ Nottingham Digestive Diseases Centre, University of Nottingham, Nottingham, United Kingdom

**Keywords:** hyoscine butylbromide, HBB, scopolamine butylbromide, drotaverine, paracetamol, peppermint oil, mechanism of action, abdominal cramping pain

## Abstract

**Introduction:**

Drotaverine, paracetamol, and peppermint oil are often prescribed for the treatment of gastrointestinal spasm and pain. This study aimed to evaluate the effect of these drugs alone and combined with the well-known antispasmodic hyoscine butylbromide on the human colon.

**Methods:**

Colon samples were obtained from macroscopically normal regions of 68 patients undergoing surgery and studied in muscle bath. Drotaverine, paracetamol, and peppermint oil were tested alone and in combination with hyoscine butylbromide on (1) spontaneous contractility induced by isometric stretch (in the presence of 1 µM tetrodotoxin) and (2) contractility induced by 10^–5^ M carbachol and after (3) electrical field stimulation-induced selective stimulation of excitatory (in the presence of 1 mM Nω-nitro-L-arginine and 10 µM MRS2179) and (4) inhibitory (under non-adrenergic, non-cholinergic conditions) pathways. (5) Drotaverine alone was also tested on cAMP-dependent pathway activated by forskolin.

**Results:**

Compared with the vehicle, drotaverine and paracetamol (10^−9^–10^−5^ M) did not modify spontaneous contractions, carbachol-induced contractions, and responses attributed to selective activation of excitatory pathways. The addition of hyoscine butylbromide (10^−7^–10^−5^ M), concentration-dependently reduced myogenic contractions and carbachol- and electrical field stimulation-induced contractile responses. The association of paracetamol (10^−4^ M) and hyoscine butylbromide (10^−7^–10^−5^ M) was not different from hyoscine butylbromide alone (10^−7^–10^−5^ M). At higher concentrations (10^−3^M–3*10^−3^ M), paracetamol decreased myogenic and carbachol-induced contractions. The adenylate cyclase activator, forskolin, concentration-dependently reduced contractility, leading to smooth muscle relaxation. The effect of forskolin 10^–7^ M was concentration-dependently enhanced by drotaverine (10^−6^M–10^−5^M).

**Discussion:**

Peppermint oil reduced myogenic activity and carbachol- and electrical field stimulation-induced contractions. The association of hyoscine butylbromide and peppermint oil was synergistic since the interaction index measured with the isobologram was lower than 1. No effect was seen on the neural-mediated inhibitory responses with any of the drugs studied although peppermint oil reduced the subsequent off-contraction. Drotaverine and hyoscine butylbromide have a complementary effect on human colon motility as one stimulates the cAMP inhibitory pathway and the other inhibits the excitatory pathway. Peppermint oil is synergic with hyoscine butylbromide suggesting that a combination therapy may be more effective in treating patients. In contrast, at therapeutic concentrations, paracetamol does not modify colonic contractility, suggesting that the association of paracetamol and hyoscine butylbromide has independent analgesic and antispasmodic properties.

## 1 Introduction

Abdominal cramping pain is a symptom in the general population. Antispasmodic medications are widely used to treat abdominal cramping pain with the objective of relaxing gastrointestinal (GI) smooth muscle. The class of antispasmodics includes (1) antimuscarinics such as hyoscine butylbromide (HBB), (2) phosphodiesterase (PDE) inhibitors such as drotaverine and papaverine, (3) sodium channel blockers such as mebeverine, (4) peripheral opiate receptor agonists such as trimebutine, (5) calcium channel blockers such as otilonium and pinaverium, (6) direct smooth muscle relaxant such as alverine, and (7) herbal medicinal products such as peppermint oil (Muller-Lissner et al., 2022). Acetaminophen (paracetamol) is also widely used as an analgesic to treat abdominal pain, but some data suggest that this could also act as an antispasmodic (Muller-Lissner et al., 2022; Mousavi et al., 2023).

Drotaverine hydrochloride is an iso-quinoline derivative that is structurally related to papaverine NLM (2023). The mechanism of action (MoA) of drotaverine is based on the inhibition of PDE4 isoenzyme, leading to smooth muscle relaxation. However, in the airway (ED_50_ = 22–44 µM) and uterine (IC_50_ = 2.6–5.6 µM) smooth muscle, it might bind to L-type calcium channels, causing further smooth muscle relaxation. No anticholinergic effects have been described in animal models (Tomoskozi et al., 2002; Patai et al., 2016; Patai et al., 2018). The effect of drotaverine on human colonic contractility has never been characterized.

Peppermint oil is a volatile oil obtained from the flowering parts and leaves of *Mentha piperita L.* (Lamiaceae). Menthol is the primary component of peppermint oil and is considered to be primarily responsible for the spasmolytic effects of the oil (Hills and Aaronson, 1991). Menthol was found to concentration-dependently (0.3–10 mM) reduce electrical field stimulation (EFS)-induced contractions and abolished carbachol (cch)-induced contractions at 1 mM (Amato et al., 2014). *In vitro* studies on guinea pig taenia coli showed that peppermint oil (88 μg/mL) reduces acetylcholine-, cch-, serotonin-, substance P- and histamine-induced contractions. The mechanism is probably related to the inhibition of L-type calcium channels (Hills and Aaronson, 1991). However, different transient receptor potential channels (TRP) such as TRPM8, TRPV_1_ and TRPA_3_ could be also involved in the response since menthol is a ligand of these receptors (Blair et al., 2023). In the study conducted by Hills and Aaronson, peppermint oil non-competitively inhibited depolarization-mediated contractions by blocking the voltage-dependent calcium channel currents in the guinea pig taenia coli; and menthol which is the major constituent is responsible for these actions (Hills and Aaronson, 1991). Strong inhibition of spontaneous contractions by peppermint and caraway oils (colon IC_50_: 17 ± 0.2 μg/mL and 61 ± 7 μg/mL, respectively) was demonstrated in the human intestine. Moreover, peppermint oil 55 μg/mL and caraway 127 μg/mL also induced epithelial secretion in the human colon (Krueger et al., 2020). Both actions were concluded to be nerve-independent and possibly mediated by inhibition of L-type calcium channels.

Paracetamol (acetaminophen) is an analgesic and antipyretic drug (Graham et al., 2013) that is often prescribed for abdominal pain (Chen et al., 2017; Muller-Lissner et al., 2022). Its MoA is related to the inhibition of cyclooxygenase-mediated production of prostaglandins and the endocannabinoid system and serotonergic pathways (Vane and Botting, 1998; Graham et al., 2013; Przybyla et al., 2020). However, it has also been demonstrated to inhibit airway smooth muscle contraction by blocking the L-type voltage-dependent calcium channel, store-operated calcium ion entry, canonical transient receptor potential-3 and 5, and calcium sensitization (Chen et al., 2020). It also showed a spasmolytic effect on rat uterine (3 and 6 mM) and aortic smooth muscle (500 mg/kg/single dose orally given) by inhibiting calcium influx (Couso et al., 1996; Correia et al., 2022). *In vitro* studies showed its effect in reducing myogenic activity and EFS-induced contractions (100–500 μmol/L) (Donnerer and Liebmann, 2017) and impairing peristalsis in guinea pig ileum at 100 µM (Herbert et al., 2005). In contrast, the effect of paracetamol on human colonic contractility has never been investigated.

HBB, also known as scopolamine butylbromide, is a tropane alkaloid and a derivative of the tertiary ammonium compound hyoscine. HBB is an antispasmodic drug, used in many countries to treat abdominal pain associated with smooth muscle spasm in both GI and genitourinary tracts (Corsetti et al., 2023). Recent evidence demonstrates that HBB (10^−7^ to 10^−5^ M) has an antimuscarinic effect that blocks acetylcholine contraction from neural and potentially non-neural origin, suggesting that HBB has spasmolytic properties regardless of the source of acetylcholine responsible for the spasm (Traserra et al., 2024).

The colon is a complex neuromuscular organ where many types of neurons, interstitial cells, and muscle fibers coexist. These different cell types subtly coordinate colonic motility, which in its entirety is poorly understood (Corsetti et al., 2019). *In vitro* studies with human tissue are essential to characterize the MoA of drugs without the need to extrapolate from experimental animal studies. HBB is available in formulation combined with paracetamol. Whether the combination of HBB with drotaverine, peppermint oil, and paracetamol can facilitate their effect as single agents is unknown. This study assessed the effects of drotaverine, paracetamol, and peppermint oil on different mechanisms affecting myogenic activity and excitatory and inhibitory neuromuscular transmission in human colonic smooth muscle alone or in combination with the muscarinic antagonist HBB to understand whether the drugs have a complementary MoA.

## 2 Materials and methods

### 2.1 Human tissue collection and preparation

Human tissue samples of the colon were obtained from patients undergoing surgery for colon cancer at Hospital Vall d’Hebron, Barcelona, Spain after they gave their informed consent (Supplementary Table S1). The sample was selected by the pathology department of the Vall d’Hebron Hospital. The sample was taken from the area macroscopically free of tumor, leaving a sufficient margin that varied depending on the surgery. Collected samples were transported in cold saline buffer to the laboratory at the Universitat Autònoma de Barcelona, Barcelona, Spain. Thereafter, samples were rinsed and placed in Krebs solution on a dissection dish and the mucosal layer was carefully separated. Muscle strips 1 cm × 0.4 cm were cut in circular and longitudinal directions. All the experimental procedures were approved by the Ethics Committee of the Universitat Autònoma de Barcelona (ethical approval code: 5604).

Circular and longitudinal muscle strips were studied in a 10-mL organ bath filled with Krebs solution at 37°C ± 1°C and bubbled with carbogen (95% O_2_ and 5% CO_2_). A tension of 4 g was applied, and tissues were equilibrated for 1 h. An isometric force transducer (Harvard VF-1) connected to an amplifier was used to record the mechanical activity. Data were digitalized (25 Hz) using DATAWIN1 software (Panlab, Barcelona, Spain) coupled with an ISC-16 analog-to-digital card installed in a computer. Grass S88 Stimulator (Grass Instruments Co., MA, USA) and Stim—Spiller II (MedLab Instruments, CO, USA) were used to deliver the pulses to the electrodes (see below). EFS was applied through two platinum electrodes placed on the support holding the tissue to study the release of inhibitory and excitatory neurotransmitters.

### 2.2 Solutions and drugs

Krebs solution containing 10.10 mM glucose, 115.48 mM NaCl, 21.90 mM NaHCO_3_, 4.61 mM KCl, 1.14 mM NaH_2_PO_4_, 2.50 mM CaCl_2_, and 1.16 mM MgSO_4_, bubbled with a mixture of 5% CO_2_:95% O_2_ (pH 7.4), was used for the experiments. The drugs used in the experiments were drotaverine, paracetamol, peppermint oil (Supplementary Table S2), HBB (all from Sanofi, France), Nω-nitro-L-arginine (L-NNA), atropine sulfate, phentolamine, tetrodotoxin (TTX) (from Sigma Chemicals, St. Louis, MO, USA), 2′-deoxy-N6-methyl adenosine 3′,5′-diphosphate tetra-ammonium salt (MRS2179) (from Merck Millipore, Darmstadt, Germany), (2-hydroxyethyl)trimethylammonium chloride carbamate (cch), propranolol and forskolin (from Tocris, Bristol, UK). Stock solutions were made by dissolving drugs in distilled water, except for L-NNA that required sonication to be dissolved in Krebs solution, and drotaverine that was dissolved in dimethyl sulfoxide (DMSO). The total volume of DMSO added in the organ bath after cuulatives curves was 10 μL, which represented 0.1% of the total volume.

### 2.3 Experimental design of the study

This study evaluated the effects of drotaverine, paracetamol, and peppermint oil alone or in combination with HBB on (1) spontaneous myogenic contractility, (2) cch (10 μM)-induced contractility, and EFS-induced neural-mediated (3) excitatory and (4) inhibitory responses. 5) The effect of drotaverine on the cyclic adenosine monophosphate (cAMP) pathway was also evaluated upon activation of adenylyl cyclase with forskolin. Vehicle (distilled water or DMSO using the same volume used in each concentration) was used as a comparator in experiments 1, 2, and 3. HBB at a concentration of 10^–7^ to 10^–5^ M was added to each of the experiments to assess a possible complementary effect. These concentrations were selected based on a recently published study (Traserra et al., 2024).

#### 2.3.1 Experiment 1: effects of drotaverine, paracetamol, and peppermint oil on myogenic contractions

The tissue samples were incubated for 20 min with TTX (1 µM) to inhibit the neuronal action potentials. This was expected to reveal the spontaneous smooth muscle activity characterized by spontaneous phasic contractions as previously demonstrated (Rae et al., 1998; Auli et al., 2008; Carbone et al., 2013). The samples were then incubated at increasing concentrations of drotaverine (10^–9^ to 10^–5^ M), paracetamol (10^–9^ to 10^–5^ M), and peppermint oil (1–1,000 μg/mL). HBB from 10^–7^ to 10^–5^ M was subsequently added as a comparator to study a possible complementary effect. For each concentration, all drugs were incubated for 5–10 min until a stable response was obtained.

#### 2.3.2 Experiment 2: effects of drotaverine, paracetamol, and peppermint oil on carbachol pre-contracted tissue

In this experiment, tissues were incubated with cch (10^–5^ M) that caused an increase in the contractile activity. A stable mechanical activity was achieved 15–20 min after incubating with cch, and samples were then incubated at increasing concentrations of drotaverine (10^–9^ to 10^–5^ M), paracetamol (10^–9^ to 10^–5^ M), and peppermint oil (1–1,000 μg/mL). HBB at a concentration range of 10^–7^ to 10^–5^ M was added to study a possible complementary effect. For each concentration, all drugs were incubated for 5–10 min until a stable response was obtained.

#### 2.3.3 Experiment 3: effects of drotaverine, paracetamol, and peppermint oil on neural-mediated excitatory responses

To evaluate the effect of excitatory neurotransmitters released by enteric neurons, the release of inhibitory neurotransmitters was inhibited by incubating the muscle strips in non-nitrergic and non-purinergic (NNNP) conditions: 1 mM of L-NNA and 10 μM of MRS2179. EFS (voltage: 30 V, pulse duration: 0.4 ms, frequency: 50 Hz, train duration: 300 ms) was applied once each 100 s and drotaverine (10^–9^ to 10^–5^ M), paracetamol (10^–9^ to 10^–5^ M), and peppermint oil (1–1,000 μg/mL) were added at increasing concentrations. HBB at 10^–7^ to 10^–5^ M was added at the end of each experiment. For each concentration, all drugs were incubated for 5–10 min until a stable response was obtained.

#### 2.3.4 Experiment 4: effects of drotaverine, paracetamol, and peppermint oil on neural-mediated inhibitory responses

To isolate the effect of inhibitory neurotransmitters released by neurons, the excitatory neurotransmitters were inhibited by incubating tissues in non-adrenergic and non-cholinergic (NANC) conditions: atropine, propranolol, and phentolamine, all at 1 µM. Then, EFS was applied at an increasing voltage (drotaverine and paracetamol: 5, 6, 7, 8, 10, and 20 V; peppermint oil: 8, 10, and 20 V), delivered at a frequency of 5 Hz and a pulse duration of 0.4 ms for 2 min. Drotaverine (10^–6^ M), paracetamol (10^–5^ M), and peppermint oil (100 μg/mL and 1,000 μg/mL) were added to study their effect on inhibitory pathway. For each concentration, all drugs were incubated for 5–10 min until a stable response was obtained.

#### 2.3.5 Experiment 5: effect of drotaverine on cAMP pathway

A concentration–response curve with the adenylate cyclase activator forskolin was obtained to determine the concentration of forskolin that reduced myogenic activity. Accordingly, the tissue was incubated with drotaverine (10^–6^, 3 × 10^−6^, and 10^–5^ M) in the presence of forskolin (10^–7^ M) or vehicle to assess a possible potentiation of drotaverine on forskolin response. For each concentration, all drugs were incubated for 5–10 min until a stable response was obtained.

### 2.4 Data analysis

The area under the curve (AUC) (g*min) of contractions from the baseline was measured to estimate the effects of drugs in different conditions. Five-minutes period was taken in the control period and after each concentration was added to assess the drug effect. To normalize mechanical data, responses were expressed as a percentage of the basal AUC using the following formula: 100 × (AUC during EFS or after drug incubation/AUC previous to EFS or drug addition). Using this formula, 0% represents the complete cessation of any activity; whereas 100% denotes no change compared with basal activity. This procedure was used for experiments 1, 2, 4, and 5, where the effect of drugs on myogenic activity, cch-induced contractions, or inhibitory pathway was tested.

For EFS-induced excitatory responses, experiment 3, and to assess the off-contraction, experiment 4, the amplitude of the response was measured before and after drug addition, data were normalized (i.e., 100% = amplitude before drug addition), and the percentage of reduction was calculated for each drug.

In the experiments performed with peppermint oil and paracetamol, when a reduction in the AUC of spontaneous phasic contractions was observed then, the amplitude, frequency of contractions, and the baseline, which is indicative of the tone, were also measured. These data were normalized and compared with the basal activity, Since both HBB and peppermint oil reduced several of the measured parameters, we evaluated their possible additive or synergistic effect by means of an isobolographic study. Accordingly, we measured the interaction index based on the previously published calculation (Chou, 2006). The interaction index calculates the possible additive, synergistic or antagonistic effect depending on whether it is less than, equal to or greater than 1, respectively (Chou, 2006).

### 2.5 Statistical analysis

Paired one-way analysis of variance (ANOVA) was used to assess the effect of drugs at different concentrations. Dunnett’s *post hoc* test was used to assess differences between groups. Paired two-way ANOVA was used to compare drug and vehicle at different concentrations or voltage of stimulation. Tukey’s *post hoc* test was used to assess differences between groups. A nonlinear regression analysis, *Y* = 100/(1 + 10^((LogIC_50_ − *X*)*Hill Slope)), was performed to assess the effect of forskolin on myogenic contractions. Graph data were expressed as the mean ± standard error of the mean (SEM) and were considered significant when *p* < 0.05. In each experimental condition, the *n* value represents the total number of muscle strips used from different patients. From each patient, a mean of about 8 strips was studied depending on the availability of the tissue. A statistical analysis was performed with GraphPad Prism version 6.01 (GraphPad Software, San Diego, CA, USA).

## 3 Results

Colon samples were obtained from macroscopically normal regions of 68 patients (26 women and 42 men, aged 35–93 years) undergoing surgery for colon cancer. The details of the colonic samples collected for each experiment are presented in Supplementary Table S1.

### 3.1 Drotaverine

#### 3.1.1 Experiments 1, 2, and 3: effect of drotaverine on spontaneous phasic contractions, cch-induced contractions, and neural-mediated excitatory responses

**FIGURE 1:**
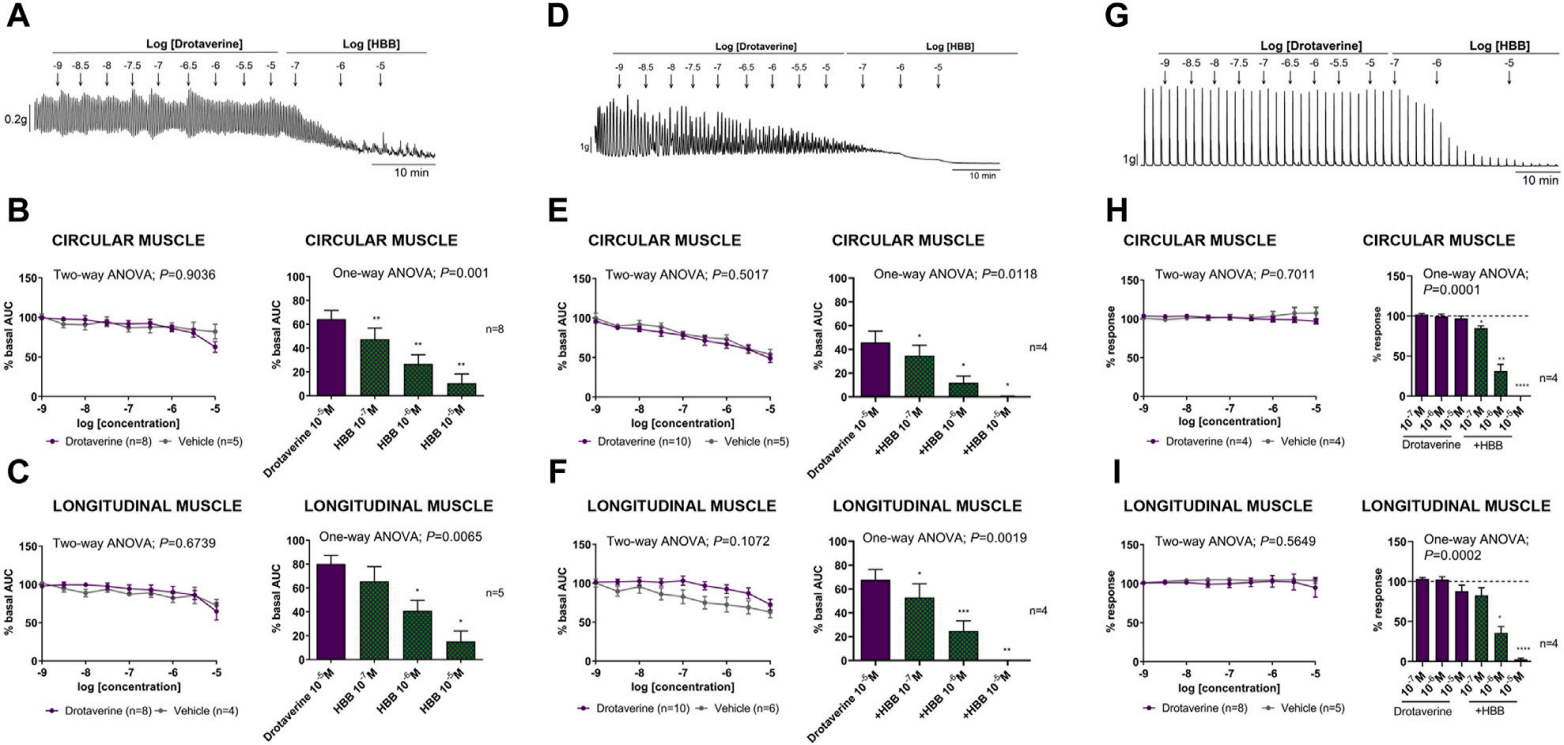
Representative mechanical recordings from the circular muscle showing the effect of drotaverine (10^–9^ to 10^–5^ M) and HBB (10^–7^ to 10^–5^ M) on spontaneous phasic contractions **(A)**, cch (10^–5^ M)-induced contractions **(D)**, and the excitatory neural pathway under non-nitrergic non-purinergic conditions **(G)**. Graphs (left) show the effect of accumulative concentrations of drotaverine (10^–9^ to 10^–5^ M). Two-way ANOVA (concentration x treatment) was performed without differences between drotaverine and vehicle (treatment *p* > 0.05). Histograms (right) show the effect of drotaverine 10^–5^ M plus HBB (10^–7^ to 10^–5^ M) on spontaneous phasic contractions, **(B,C)** cch-induced contractions **(E,F)**, and electrical field stimulation-induced contractions **(H,I)** in the circular **(B,E,H)** and longitudinal **(C,F,I)** muscles. Dunnett’s *post hoc* test was done after ANOVA. *p < 0.05, **p < 0.01, ***p < 0.001, ****p < 0.0001 compared with drotaverine 10^–5^ M. Data were normalized (i.e., 100%) to the basal AUC or the electrical field stimulation amplitude before drug addition. Data were expressed as mean ± SEM.

Figure 1 shows the effect of drotaverine as compared with the vehicle (DMSO) on spontaneous phasic contractions, cch-induced contractions, and neural-mediated excitatory responses of circular and longitudinal muscles. In the presence of the neural blocker TTX, both circular and longitudinal muscles displayed spontaneous contractions. Drotaverine up to 10^–5^ M did not affect spontaneous contractions compared with the vehicle. The addition of HBB (10^–7^ to 10^–5^ M) in the presence of drotaverine, concentration-dependently reduced myogenic contractions both in the circular and longitudinal layers (Figures 1A–C).

The muscarinic agonist, cch, induced an increase in contractions in both the circular and longitudinal muscles (not shown). The effect of drotaverine in comparison with the vehicle alone and in the presence of HBB is shown in Figures 1D, E, F. Drotaverine did not modify cch-induced contractions compared with the vehicle in the circular or longitudinal muscle. However, the subsequent addition of HBB (10^–7^ to 10^–5^ M) totally abolished the cholinergic response.

In the presence of L-NNA 1 mM and MRS2179 10 μM, EFS induced a sharp contraction associated with selective activation of excitatory motor neurons. The effect of drotaverine in comparison with the vehicle alone or in the presence of HBB on neural-induced excitatory responses is shown in Figures 1G, H, I. In both muscle layers, drotaverine did not modify EFS-induced contractions compared with the vehicle. The subsequent addition of HBB (10^–7^ to 10^–5^ M) totally abolished neural-mediated excitatory responses.

#### 3.1.2 Experiment 4 and 5: effect of drotaverine on neural-mediated inhibitory responses and cAMP-dependent pathway

**FIGURE 2:**
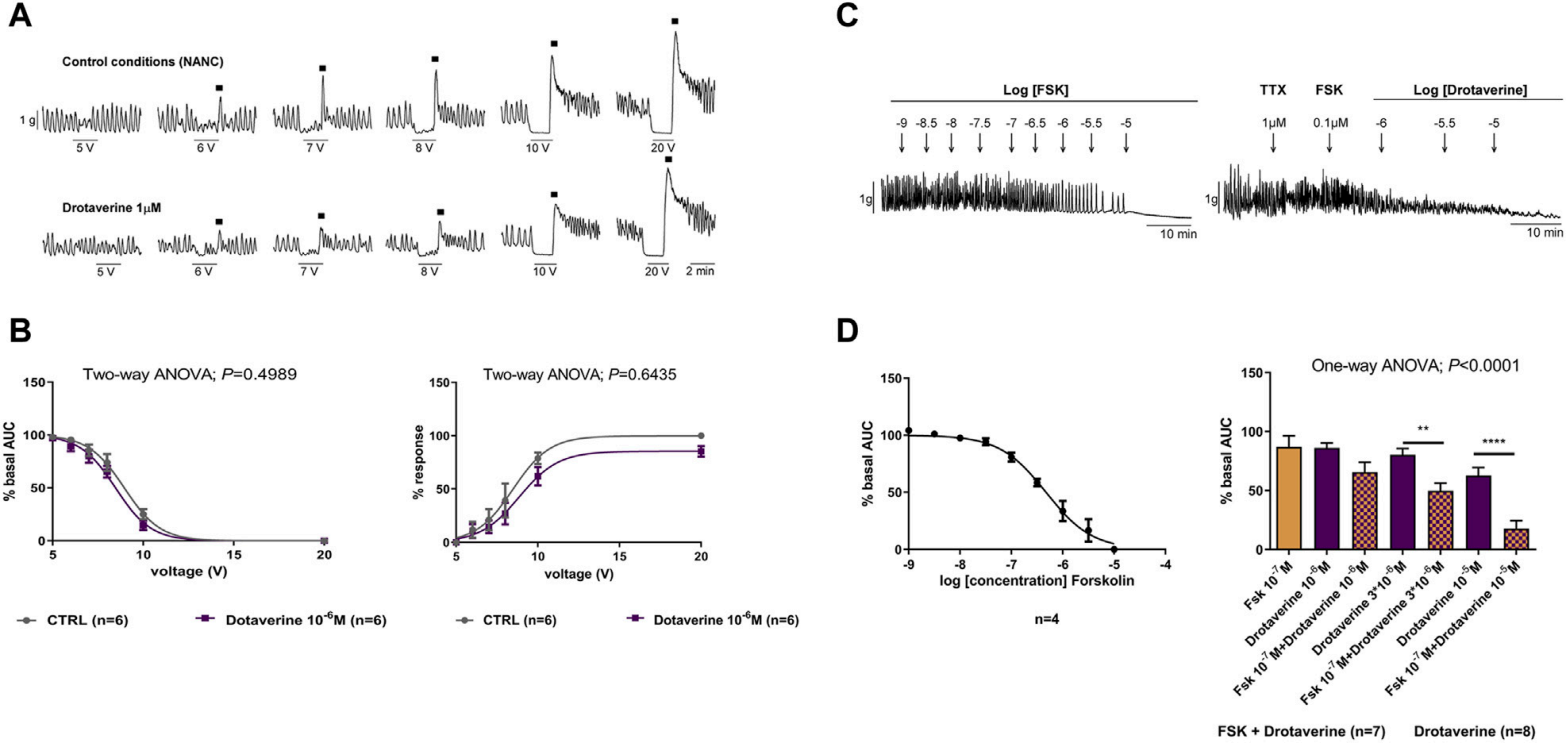
The effect of drotaverine on neural-mediated inhibitory responses under non-adrenergic non-cholinergic (NANC) conditions and on the degradation of the cAMP in the circular muscle. Representative mechanical recording from the circular muscle showing the relaxation (during the electrical field stimulation) and the off-contraction (rebound after the electrical field stimulation) in control conditions and in the presence of drotaverine 10^–6^ M **(A)**. Graphs show the effect of drotaverine 10^–6^ M on electrical field stimulation trains of increasing voltage (5, 6, 7, 8, 10 and 20 V), on the relaxation (left), and the off-contraction (right) **(B)**. Two-way ANOVA (voltage x treatment) was performed without differences with or without drotaverine (treatment *p* > 0.05). Relaxation was normalized (i.e., 100%) to the basal AUC before electrical field stimulation. Off-contraction was normalized (i.e., 100%) to the maximum amplitude response obtained with a stimulus of 20 V in control conditions. Representative mechanical recording from the circular muscle shows the effect of forskolin (FSK) (left) and the effect of FSK 10^–7^ M plus drotaverine 10^–6^, 3 × 10^−6^, and 10^–5^ M on the spontaneous phasic contractions (right) **(C)**. Concentration–response curve of FSK (left) and histogram (right) show the effect of drotaverine on the degradation of the cAMP **(D)**. Dunnett’s *post hoc* test was done after ANOVA to compare the effect of drotaverine with and without FSK, **p < 0.01, ****p < 0.0001. Data were compared with the basal AUC before drug addition. Data were expressed as mean ± SEM.

Under NANC conditions, EFS induced a relaxation (on-relaxation), followed by a contraction (off-contraction) just after the end of the stimulus. Both the relaxation and the off-contraction were voltage-dependent (Figures 2A, B). These responses are normally abolished by tissue incubation with L-NNA 1 mM and MRS2179 10 µM (Gallego et al., 2008; Traserra et al., 2024), suggesting that both on-relaxations and off-contractions are associated to the release of nitric oxide (NO) and adenosine triphosphate (ATP) by inhibitory neurons. Figures 2A, B shows that drotaverine 10^–6^ M did not modify neural-mediated inhibitory response since neither the relaxation nor the off-contraction was different compared with the control.

The adenylate cyclase activator, forskolin (10^–9^ to 10^–5^ M), concentration-dependently inhibited spontaneous phasic contractions (LogIC_50_ = −6.33 ± 0.054) (Figures 2C, D). The concentration of 10^–7^ M was selected to test a possible enhancement of drotaverine on forskolin responses. At 10^–7^ M, the effect of forskolin was very mild (inhibition of 19.23% ± 3.87%). When forskolin 10^–7^ M was incubated with increasing concentrations of drotaverine (10^–6^, 3 × 10^−6^, and 10^–7^ M), the relaxation induced by forskolin was increased (Figures 2C, D). These experiments were performed in the presence of TTX to avoid potential influences of neural activities on smooth muscle responses.

### 3.2 Paracetamol

#### 3.2.1 Experiments 1, 2, 3, and 4: effect of paracetamol on spontaneous phasic contractions, cch-induced contractions, and neural-mediated excitatory and inhibitory responses

**FIGURE 3:**
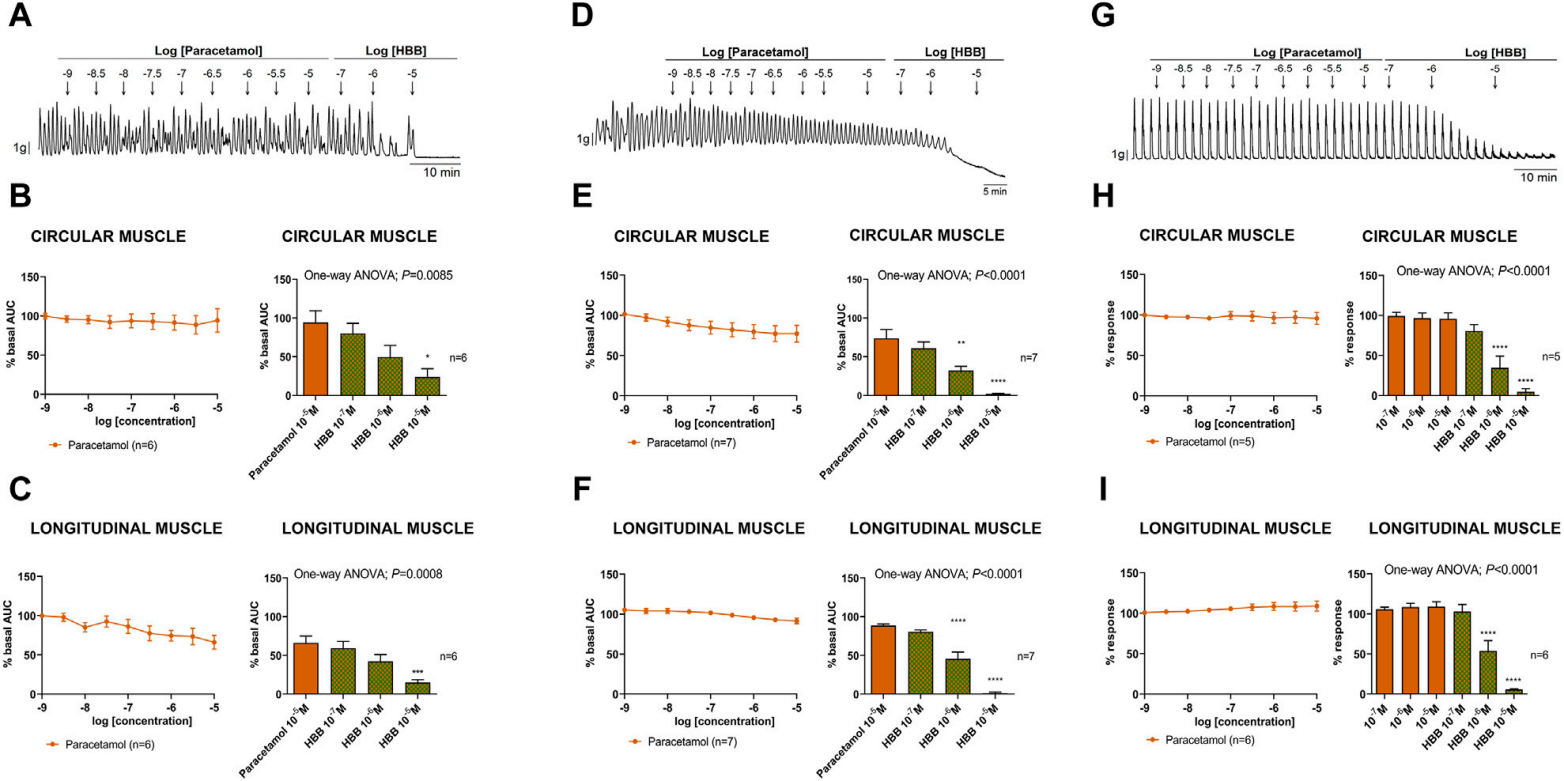
Representative mechanical recordings from the circular muscle show the effect of paracetamol (10^–9^ to 10^–5^ M) and HBB (10^–7^ to 10^–5^ M) on spontaneous phasic contractions **(A)**, cch (10^–5^ M)-induced contractions **(D)**, and the excitatory neural pathway under non-nitrergic non-purinergic conditions **(G)**. Graphs (left) show the effect of accumulative concentrations of paracetamol (10^–9^ to 10^–5^ M) andhistograms (right) show the effect of paracetamol 10^–5^ M plus HBB (10^–7^ to 10^–5^ M) on spontaneous phasic contractions **(B,C)**, cch-induced contractions **(E,F)**, and electrical field stimulation-induced contractions **(H,I)** in the circular **(B,E,H)** and longitudinal **(C,F,I)** muscles. Dunnett’s *post hoc* test was done after ANOVA. *p < 0.05, **p < 0.01, ***p < 0.001, ****p < 0.0001 compared with paracetamol 10^–5^ M. Data were normalized (i.e., 100%) to the basal AUC or the electrical field stimulation amplitude before drug addition. Data were expressed as mean ± SEM.

**FIGURE 4:**
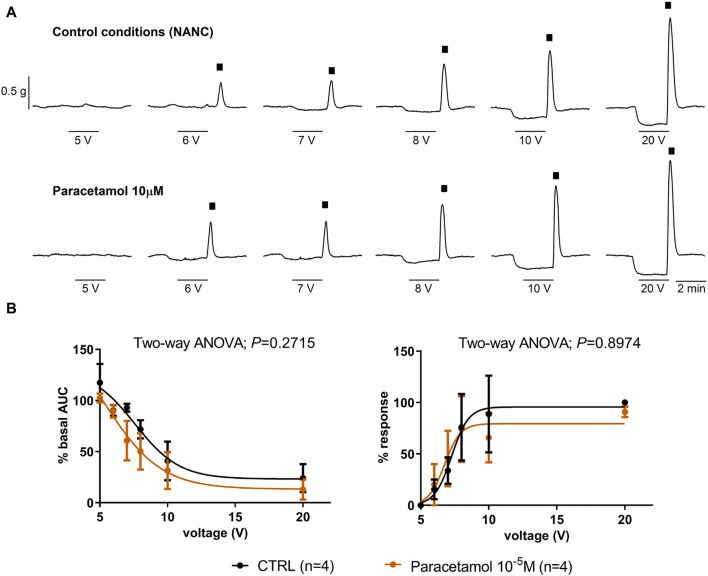
The effect of paracetamol on neural-mediated inhibitory responses under non-adrenergic non-cholinergic conditions in the circular muscle. Representative mechanical recordings show the relaxation (during the electrical field stimulation) and the off-contraction (rebound after the electrical field stimulation) in control (CTRL) conditions (top) and in the presence of paracetamol 10^–5^ M (bottom) **(A)**. Graphs show the effect of paracetamol 10^–5^ M on electrical field stimulation trains of increasing voltage (5, 6, 7, 8, 10, and 20 V) on the relaxation (left) and the off-contraction (right) **(B)**. Two-way ANOVA (voltage x treatment) was performed without differences with or without paracetamol 10^−5^ M (treatment *p* > 0.05). The relaxation was normalized (i.e., 100%) to the basal AUC before electrical field stimulation. The off-contraction was normalized (i.e., 100%) to the maximum response obtained with a stimulus of 20 V in CTRL conditions. Data were expressed as mean ± SEM.

Paracetamol at a range of concentrations from 10^–9^ to 10^–5^ M did not reduce spontaneous phasic contractions, cch-induced contractions, and EFS-induced contractions. In contrast, the addition of HBB (10^–7^ to 10^–5^ M), in the presence of paracetamol, concentration-dependently reduced all the responses (Figure 3). Paracetamol at 10^–5^ M did not modify neural-mediated inhibitory responses as both on-relaxations and off-contractions were not different from control EFS (Figure 4).

#### 3.2.2 Experiments 1, 2 and 3 with higher concentrations of paracetamol

**FIGURE 5:**
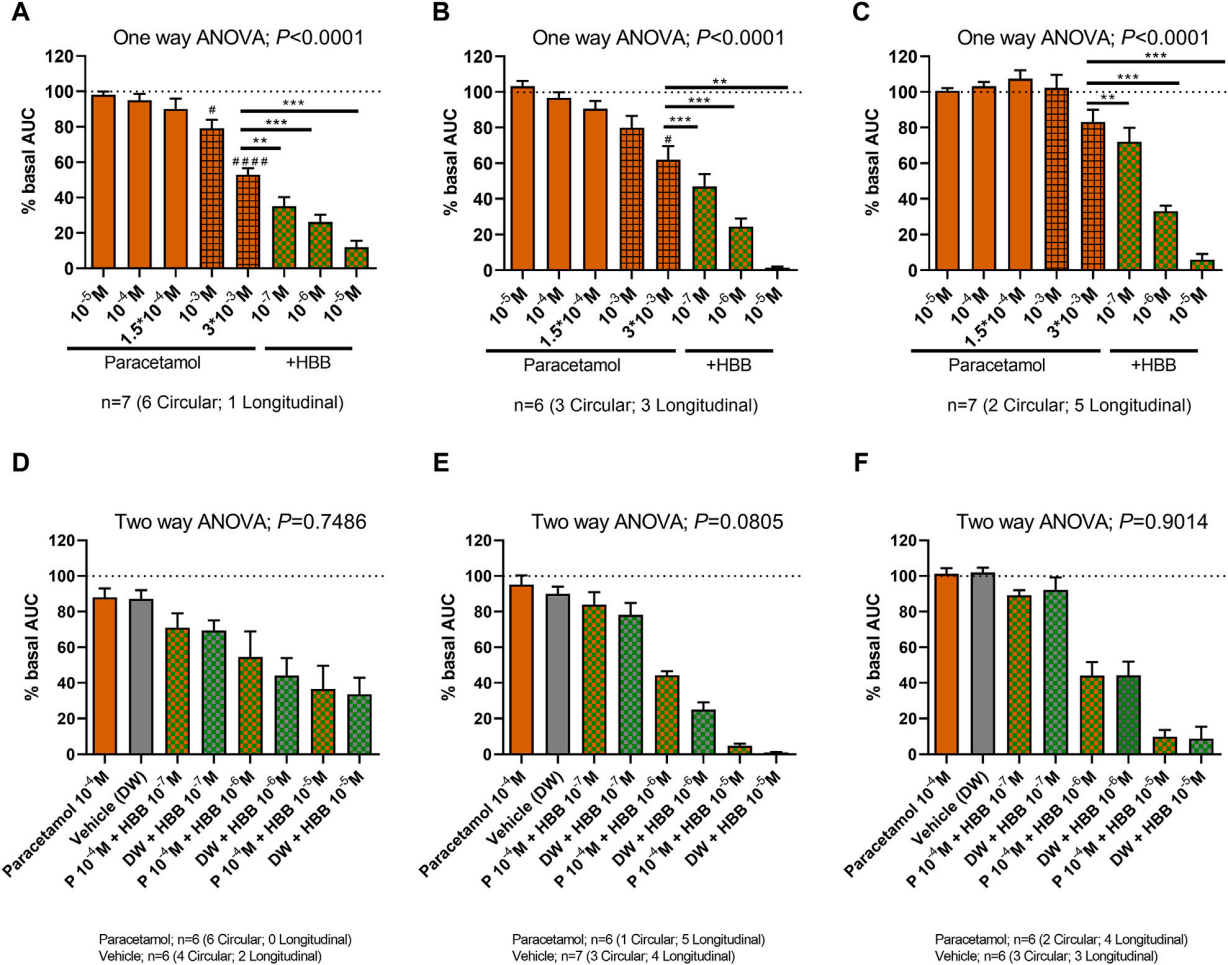
The effect of paracetamol on spontaneous phasic contractions, cch-induced contractions, and excitatory electrical field stimulation. (Top) Histograms show the effect of paracetamol (10^–5^ M to 3 × 10^−3^ M) plus HBB (10^–7^ to 10^–5^ M) on spontaneous phasic contractions **(A)**, cch-induced contractions **(B)**, and excitatory electrical field stimulation **(C)**. Dunnett’s *post hoc* test was done after ANOVA. ^#^ *p* < 0.05, ^####^ *p* < 0.0001 compared with basal conditions and *p < 0.05, **p < 0.01, ***p < 0.001, ****p < 0.0001 compared with paracetamol 3 mM **(A–C)**. (Bottom) Histograms show the effect of paracetamol (100 µM) plus HBB (10^–7^ to 10^–5^ M) compared with HBB (10^–7^ to 10^–5^ M) plus vehicle on spontaneous phasic contractions **(D)**, cch-induced contractions **(E)**, and excitatory electrical field stimulation **(F)**. Two-way ANOVA (concentration x treatment) was performed to test a potential complementary effect of paracetamol and HBB. Notice that although a concentration dependent reduction was observed in both groups (concentration: *p* < 0.0001) the combination of paracetamol and HBB was not different from the effect of HBB plus vehicle (treatment *p* > 0.05). Data were normalized (i.e., 100%) to the basal AUC or the electrical field stimulation amplitude before drug addition. Data were expressed as mean ± SEM.

Since no effect of paracetamol was seen at concentrations below 10^–5^ M, the experiments were repeated mimicking the maximum concentrations of the drug achieved in plasma (Forrest et al., 1982; Bannwarth et al., 1992; Tanner et al., 2010). Three concentrations of the drug that are associated with therapeutic concentrations (10^–5^, 10^–4^ and 1.5 × 10^−4^ M) and two supratherapeutic concentrations (10^–3^ and 3 × 10^−3^ M) were used, the latter clearly above the concentrations found in plasma (Forrest et al., 1982; Bannwarth et al., 1992; Tanner et al., 2010). At therapeutic concentrations, paracetamol did not modify spontaneous phasic contractions, cch-induced contractions, and EFS-induced contractions. However, at supratherapeutic concentrations, a partial decrease in spontaneous motility and cch-induced contractions was observed (Figures 5A–C). The reduction in AUC of spontaneous contractions was mainly due to a reduction in amplitude (3 × 10^-3^ M) and frequency (10^−3^ and 3 × 10^-3^ M). It is important to note that the previous concentrations tested (10^−5^ to 1.5 × 10^-4^ M) did not modify the amplitude, frequency and tone (Supplementary Figure S1). The addition of HBB (10^–7^ to 10^–5^ M) totally abolished all responses.

The association of paracetamol at maximum therapeutic concentration (10^–4^ M) and HBB was not different from HBB alone (plus the paracetamol vehicle: distilled water) (Figures 5D–F).

### 3.3 Peppermint oil

#### 3.3.1 Experiments 1, 2, and 3: effect of peppermint oil on spontaneous phasic contractions and cch- and EFS-induced contractions

**FIGURE 6:**
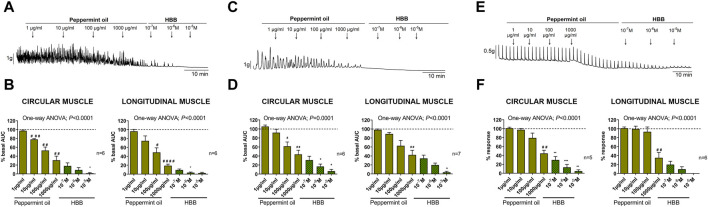
Representative mechanical recordings from the circular muscle showing the effect of peppermint oil (1–1,000 μg/mL) and HBB (10^–7^ to 10^–5^ M) on spontaneous phasic contractions **(A)**, cch (10^–5^ M)-induced contractions **(C)**, and the excitatory neural pathway under non-nitrergic non-purinergic conditions **(E)**. Histograms show the effect of peppermint oil (1–1,000 μg/mL) plus HBB (10^–7^ to 10^–5^ M) on spontaneous phasic contractions **(B)**, cch-induced contractions **(D)**, and electrical field stimulation-induced contractions **(F)** in the circular (left) and longitudinal (right) muscles. Data were normalized (i.e., 100%) to the basal AUC or the electrical field stimulation amplitude before drug addition. Dunnett’s *post hoc* test was done after ANOVA. ^#^ *p* < 0.05, ^##^ *p* < 0.01, ^###^ *p* < 0.001, ^####^ *p* < 0.0001 compared with basal conditions and *p < 0.05, **p < 0.01, ***p < 0.001, ****p < 0.0001 compared with peppermint oil 1,000 μg/mL. Data were expressed as mean ± SEM.

Peppermint oil concentration-dependently reduced the AUC of spontaneous phasic contractions both in circular and longitudinal layers. This response was mainly due to a reduction in the amplitude and frequency of contractions in both muscle layers. A slight reduction in tone was also observed in the circular muscle (Supplementary Figure S1). The addition of HBB in the presence of peppermint oil 1,000 μg/mL further reduced these contractions (Figures 6A, B). Peppermint oil also reduced, in a concentration-dependent manner, cch-induced contractions (Figures 6C, D) and EFS-induced excitatory responses (Figures 6E, F) in both muscle layers. Further addition of HBB totally abolished both responses.

#### 3.3.2 Experiment 4: effect of peppermint oil on neural-mediated inhibitory responses

**FIGURE 7:**
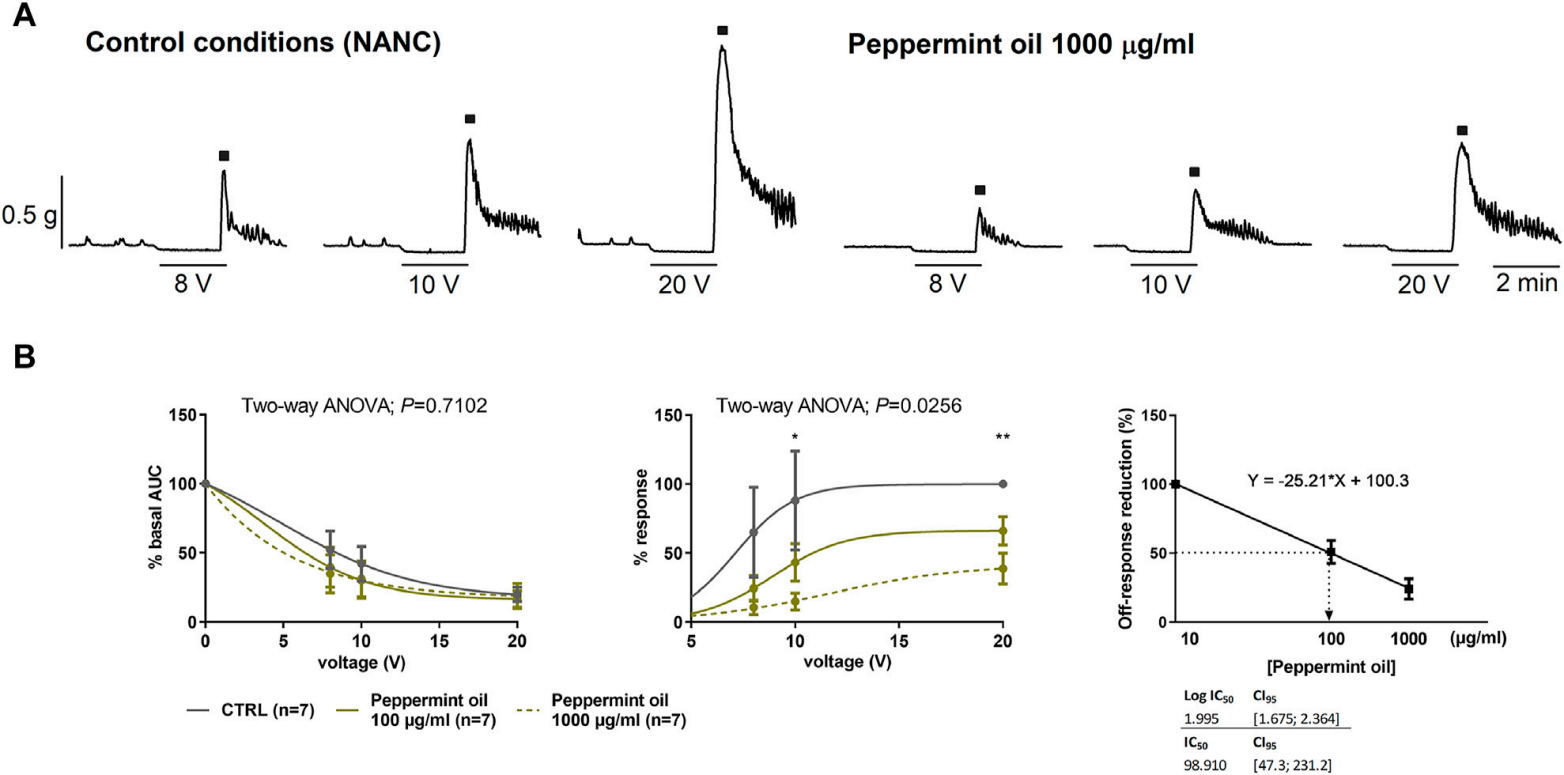
The effect of peppermint oil on neural-mediated inhibitory responses under non-adrenergic non-cholinergic (NANC) conditions in the circular muscle. Representative mechanical recordings show the relaxation (during the electrical field stimulation) and the off-contraction (rebound after the electrical field stimulation) in control (CTRL) conditions (left) and in the presence of peppermint oil 1,000 μg/mL (right) **(A)**. Graphs show the effect of peppermint oil on electrical field stimulationtrains of increasing voltage (8, 10, and 20 V) on the relaxation and the off-contraction **(B)**. Two-way ANOVA (voltage x treatment) was performed to assess the response of peppermint oil compared to the control in the on-relaxation (treatment *p* > 0.05) and the off-contraction (treatment *p* < 0.0256). Tukey’s *post hoc* test was done to compare the effect of peppermint oil with control in the off-contraction, *p < 0.05, **p < 0.01. The relaxation was normalized (i.e., 100%) to the basal AUC before electrical field stimulation. The off-contraction was normalized (i.e. 100%) to the maximum response obtained with a stimulus of 20 V in control conditions. The reduction of the off-contraction (%) was calculated through a linear regression where Y represents the Off-response reduction and X is the slope of the linear regression. The IC_50_ of this effect was about 100 μg/mL. Data were expressed as mean ± SEM.

Two concentrations of peppermint oil (100 and 1,000 μg/mL) were used to assess its effect on neural-mediated inhibitory responses. No major effect was seen on the on-relaxation, suggesting no change in the release and post-junctional responses of inhibitory neurotransmitters. In contrast, a strong decrease in the off-contraction was clearly observed (Figures 7A, B). The IC_50_ of this effect was about 100 μg/mL (Figure 7B). It is important to note that off-contractions were independent of muscarinic receptors as NANC conditions were used in these experiments.

**FIGURE 8:**
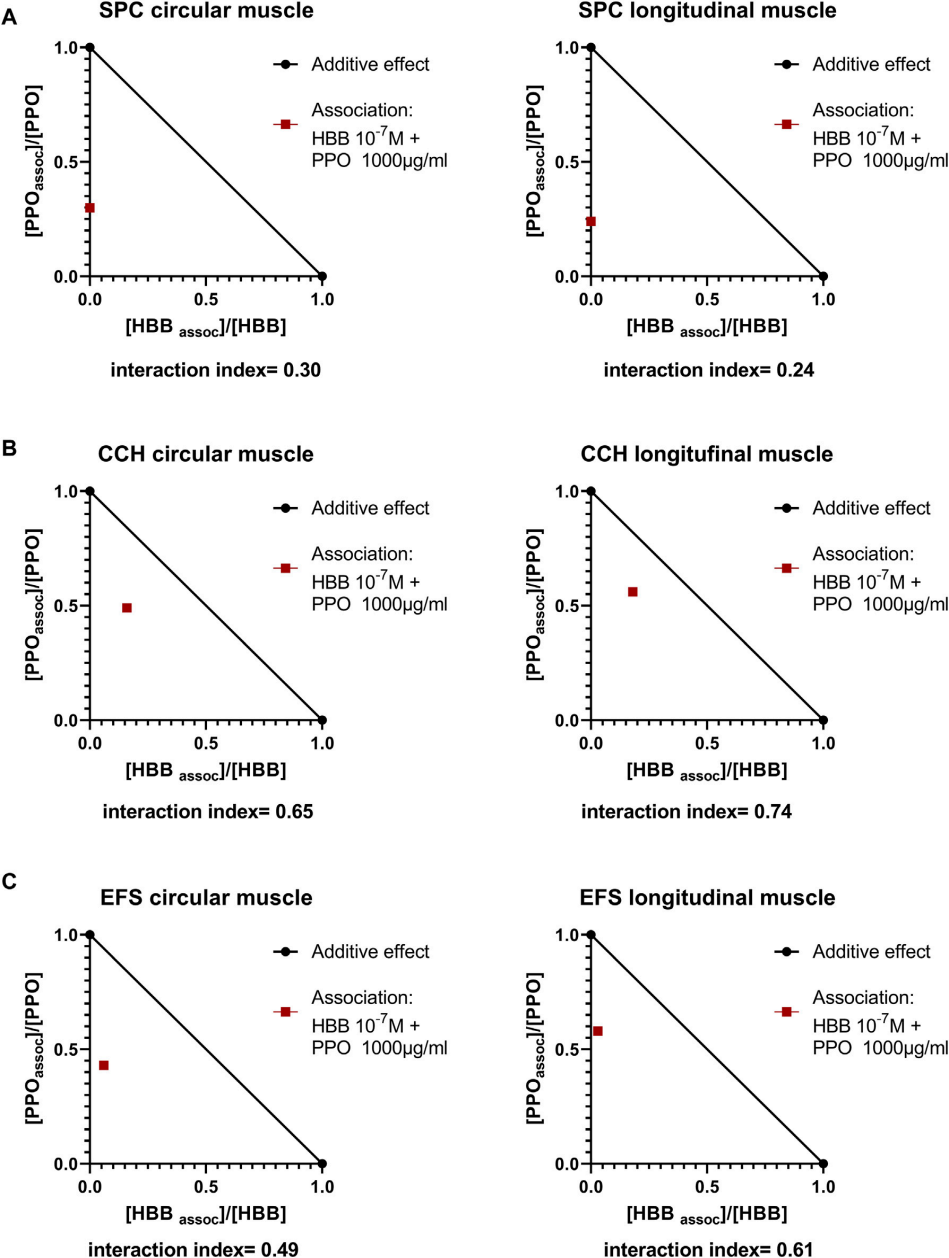
Normalized isobolograms showing the interaction index calculated with the association of HBB 10^−7^ M and peppermint oil (PPO) 1000 μg/mL **(A)**. Spontaneous phasic contractions (SPC) **(B)**, Carbachol (cch)-induced contractions and **(C)** Electrical field stimulation (EFS)-induced contractions. To build the isobologram, data for PPO (alone) were obtained with concentration-response curves (present study), and for HBB (alone) data were obtained from concentration-response curves from our previous study (Traserra et al., 2024). Interactions index between 0.1 and 0.3 are considered strong synergism, between 0.3 and 0.7 synergism, and between 0.7–0.85 moderate synergism (Chou, 2006).

Since both peppermint oil (present manuscript) and HBB (Traserra et al., 2024) reduced spontaneous phasic contractions, cch- and EFS-induced contractions we calculated the interaction index between these two drugs (Figure 8). We estimated the interaction index calculated with the mean effect of the combination of peppermint oil 1000 μg/mL and HBB 10^−7^ M. The estimated interaction index was lower than 1 in all parameters measured and the strongest synergism found occurred for spontaneous contractions in both muscle layers (Figure 8) which is consistent with a synergistic effect between HBB and peppermint oil.

## 4 Discussion

This is the first ever comprehensive assessment of the effect of drotaverine, paracetamol, and peppermint oil each alone and in combination with HBB on neuromuscular response, performed with the same protocol including selectively stimulated excitatory and inhibitory responses in the human intestine *ex vivo*. It is important to mention that neural-mediated excitatory responses were isolated incubating the tissue with L-NNA and MRS2179. Neural-mediated excitatory responses are cholinergic since they are concentration-dependently reduced by HBB and atropine (Traserra et al., 2024). In contrast, inhibitory responses were studied in NANC conditions. The inhibitory response consists of a neural release of NO and a related purine followed by an off-contraction recorded after the end of the stimulus (Broad et al., 2019). In NANC conditions, the off-contraction is independent of the activation of muscarinic receptors, and it is strongly inhibited by L-NNA and MRS2179 suggesting that this off-contraction is due to the depolarization of the smooth muscle after the inhibitory process (Traserra et al., 2024).

### 4.1 Drotaverine

The results show that compared with the vehicle, drotaverine did not modify spontaneous phasic or myogenic contractions, cch-induced contractions, and responses attributed to selective activation of excitatory pathways in both circular and longitudinal muscle layers. In contrast, the subsequent addition of HBB (10^–7^ to 10^–5^ M) on top of drotaverine concentration-dependently reduced all these contractile responses. This effect is probably due to HBB and not to drotaverine (Traserra et al., 2024). The EFS-induced relaxation, which is mediated by NO and ATP, was neither modified by drotaverine nor HBB. However, drotaverine enhanced the smooth muscle relaxation induced by the adenylate cyclase activator, forskolin 10^–7^ M.

Previous studies have reported that the PDE4 inhibitors, rolipram and roflumilast, produced a dose-related inhibition of stress-induced increased fecal pellet output *in vivo* in animals (Barone et al., 2008; Yuan et al., 2022). Moreover, high concentrations of rolipram (higher than 10^–5^ M) inhibited the spontaneous contraction of colonic smooth muscle strips *in vitro*, and this inhibitory effect was partly through the cAMP–-PKA-p-CREB pathway and NO pathway (Yuan et al., 2022). In the present study, the PDE4 inhibitor drotaverine did not affect the spontaneous activity of the smooth muscle. This suggests that cAMP is not endogenously produced in human tissue without external activation of the adenylate cyclase.

5-hydroxytryptamine receptor 4 (5-HT4) agonists, such as prucalopride, caused the facilitation of acetylcholine release from enteric motor neurons, leading to an increase in smooth muscle contraction. This is the MoA of some prokinetic drugs. The signal transduction of 5-HT4 receptors on the cholinergic nerves toward the circular muscle layer is regulated by PDE4 in the pig colon (Priem et al., 2013) and human large intestine (Pauwelyn et al., 2018). Through this, it has been shown that PDE4 inhibitors enhance the release of acetylcholine (Pauwelyn et al., 2018). An increase in neural-mediated excitatory response was observed after PDE4 inhibition with rolipram. It was proposed that an association of a 5-HT4 agonist with a PDE4 inhibitor might enhance the prokinetic effect of 5-HT4 agonists. In the present study, the PDE4 inhibitor, drotaverine, alone did not enhance neural-mediated excitatory responses. Further studies combining 5-HT4 agonists with drotaverine are needed to check if this drug might increase neural-mediated cholinergic responses. Smooth muscle is controlled by several inhibitory pathways leading to smooth muscle relaxation. NO and purines, acting on purinergic receptors (P2Y_1_), are the major contributors to colonic relaxation (Gallego et al., 2006; Gallego et al., 2008). NO activates guanylyl cyclase, leading to an increase in cyclic guanosine 3′,5′-monophosphate, and P2Y_1_ receptors activate sK(Ca). None of these pathways causes the activation of cAMP in smooth muscle cells. The effect of drotaverine on neural-mediated inhibitory responses was assessed, but no major effects were observed. This is consistent with a lack of effect of drotaverine on the PDE5, which is the PDE responsible for the degradation of cyclic guanosine 3′,5′-monophosphate (Al-Shboul et al., 2013).

Inhibitory neurons may also release vasoactive intestinal peptide (VIP)/pituitary adenylate cyclase-activating peptide that may participate in some circumstances on neural-mediated responses. It is well established that VIP receptors are present in smooth muscle cells and Interstitial cells of Cajal of the human colon, and this is a pathway that may be activated by VIP released from enteric neurons (Rettenbacher and Reubi, 2001). VIP/pituitary adenylate cyclase-activating peptide receptors are G-coupled receptors that activate adenylate cyclase, leading to an increase in cAMP. Forskolin is an adenylate cyclase activator that causes smooth muscle relaxation. The results of this study showed that in the presence of a low concentration of forskolin (10^–7^ M), drotaverine enhances relaxation. It is important to note that this effect was observed in the micromolar range. Accordingly, this work showed for the first time in the human colon that drotaverine increases smooth muscle relaxations associated to the cAMP-dependent pathway. A similar experimental procedure was previously reported to assess the effect of rolipram (a PDE4 inhibitor). The association of forskolin and rolipram synergistically increased cAMP content in canine colonic smooth muscle cells but did not increase cyclic guanosine 3′,5′-monophosphate content (Barnette et al., 1993). This is consistent with the above-mentioned functional experiments measuring contractility in human smooth muscle strips.

Drotaverine has moderate absorption and a slightly variable bioavailability after its oral administration. Plasma concentration of drotaverine after oral (3.4 × 10^−7^ to 1.0 × 10^−6^ M) and intravenous (2.5 × 10^−6^ M) administration of 80 mg of the drug is in the same order of magnitude as the concentrations used in the organ bath in the present work (Bolaji et al., 1996).

In contrast to drotaverine, HBB was able to reduce spontaneous contractions, cch-induced contractions, and neural-mediated excitatory responses (Traserra et al., 2024). This study shows that these effects are complementary to those of drotaverine, suggesting that a possible combination of two medications could have an effect on both excitatory and inhibitory pathways. Whether this could translate in a different effect in humans *in vivo* is an intriguing question. Interestingly, the PDE4 inhibitor, rolipram, has also shown efficacy in the treatment and prevention of experimental colitis in rodents (Hartmann et al., 2000; Videla et al., 2006).

### 4.2 Paracetamol

This study is the first to examine the possible effect of paracetamol on the human colon. In this study, paracetamol did not reduce spontaneous phasic contractions, cch-induced contractions, or neural-mediated contractions at concentrations ranging from 10^–9^ M to 1.5 × 10^−4^ M. In contrast, the addition of HBB in the presence of paracetamol, concentration-dependently reduced myogenic contractions and abolished cch-induced contractions, and neural-mediated contractions. However, this effect is probably due to HBB and not to paracetamol (Traserra et al., 2024).

A study that tested the effect of paracetamol on the peristaltic reflex in the guinea pig ileum (Herbert et al., 2005) showed that the peristaltic pressure threshold had increased at a concentration of 10^–5^ M and had produced a transient (15 min) impairment at 10^–4^ M level. The mechanism by which paracetamol impairs peristalsis appears to be independent of nitrergic neurons, and it is speculated that it may involve endogenous opioids. In another study performed in longitudinal muscle-myenteric plexus strips from guinea pig ileum, paracetamol (100–500 μmol/L) and dipyrone (metamizole, 100–500 μmol/L) reduced both cholinergic and myogenic contractions to the same extent; the authors concluded that their action on intestinal smooth muscle can be considered myogenic spasmolytic in nature (Donnerer and Liebmann, 2017). In the first set of experiments of this study, authors were not able to observe a decrease in neural-mediated excitatory responses at concentrations up to 10^–5^ M, which are slightly lower than the range of concentration (10^–5^ to 5 × 10^−4^ M) used in these studies performed in guinea pig ileum.

The MoA of paracetamol has been studied in other tissues. At a high concentration, paracetamol was able to inhibit potassium chloride-induced contractions in the rat uterus (Couso et al., 1996) (between 10^–3^ M and 10^–2^ M) and cause smooth muscle relaxation of the respiratory tract of mice (IC_50_ of 1.71 mg/mL) (Chen et al., 2020), suggesting a possible effect through calcium channels (Couso et al., 1996). However, the concentrations needed to achieve this effect were higher than the maximum concentrations used in the first set of experiments in the present study (10^–5^ M).

With doses up to 650 mg in human, the peak plasma concentrations of paracetamol achieved following administration are from 5 to 20 μg/mL. The time to reach the peak effect is 1–3 h and the duration of action is 3–4 h (data obtained from DrugBank, acetaminophen monograph, suppository). These concentrations are in the range of 33–132 μM, similar to those tested in the present study. It is important to note that only a small proportion (10%–20%) of paracetamol is bound to serum proteins and therefore the plasma concentrations measured will be very close to the free fraction of paracetamol (Milligan et al., 1994).

Based on the results of this study and in search of the spasmolytic effect reported in the literature, it was decided to perform further experiments by increasing the concentration of paracetamol. Three therapeutic concentrations (10^–5^, 10^–4^, and 1.5 × 10^−4^ M) and two supratherapeutic concentrations (10^–3^ M and 3 × 10^−3^ M) were assessed. Interestingly, at therapeutic concentrations, paracetamol did not modify myogenic activity, cch-induced contractions, and EFS-induced contractions. However, when the concentrations were increased to supratherapeutic levels, a partial decrease in all three responses was observed. When supratherapeutic concentrations are reached, the decreased response could be attributed to a partial inhibition of the L-type calcium channel, as has been described in other tissues studied (Couso et al., 1996; Chen et al., 2020). Consistent with a dependence on the muscarinic receptor, HBB further decreased all three responses. The association of maximum therapeutic concentrations of paracetamol (10^–4^ M) and HBB was not different from HBB alone (plus the paracetamol vehicle: distilled water). This suggests no synergistic effect when both drugs are combined. Based on these results, if local GI concentrations are equivalent to the concentrations observed in the plasma, it can be concluded that paracetamol does not modify myogenic activity, cch-induced contractions, or neural-mediated excitatory responses, suggesting a lack of effect of paracetamol on basic mechanisms responsible for GI motility. These results suggest that both drugs are targeting different mechanisms. HBB reduces pain by its spasmolytic effect, and paracetamol has no apparent effect on colonic motility but has analgesic properties. Paracetamol is specifically a useful analgesic partner to HBB and other antispasmodics to collectively treat abdominal cramping pain as it is known to be well tolerated in the GI tract (Jozwiak-Bebenista and Nowak, 2014).

### 4.3 Peppermint oil

Peppermint oil concentration-dependently reduced myogenic contractions in the presence of TTX 1 μM, both in circular and longitudinal layers. Consistent with this result, it has been previously reported that peppermint oil and its principal constituent, menthol, reduce the motility of the human small intestine and colon due to a direct action on smooth muscle cells (Amato et al., 2014; Krueger et al., 2020). In a study by Kruger et al., when peppermint oil was tested at a concentration of 55 μg/mL, it caused muscle relaxations (Krueger et al., 2020). The addition of HBB in the presence of peppermint oil at 1,000 μg/mL accentuated the reduction of myogenic contractions in both muscle layers. This is consistent with the dependence of acetylcholine on a non-neural input in the development of myogenic contractions.

Similarly, peppermint oil reduced cch-induced contractions, and the subsequent addition of HBB totally abolished the muscarinic response in both muscle layers. This result is in agreement with previously reported data that menthol also reduces cch-evoked contractions in the human colon (Amato et al., 2014).

Finally, peppermint oil at 1,000 μg/mL reduced neural-mediated excitatory responses both in longitudinal and circular layers of the human colon, and the subsequent addition of HBB potentiated the reduction. This is the first study to apply a selective protocol for stimulation of excitatory or inhibitory neurons using EFS to examine the MoA of peppermint oil. Peppermint oil did not modify neural-mediated inhibitory responses but reduced off-contractions. In the other study, EFS-induced contractions decreased with menthol (Amato et al., 2014).

The composition of peppermint oil is complex, and the main component is menthol (43%). The profile of the results of this study is similar to that previously described by Amato et al. (Amato et al., 2014). It is quite possible that one or more of the components of peppermint oil (including menthol) act at the level of calcium channels (Hills and Aaronson, 1991; Amato et al., 2014) to cause the reduction of myogenic activity, with a partial reduction of muscarinic activity and a reduction of the off-contraction after the inhibitory process. It has been reported that the effect of menthol could be mediated by the TRP-melastatin 8 (TRPM8) channel (Peier et al., 2002). However, a previous study demonstrated that the decrease in contractility in the human colon was not due to TRPM8 activation (Amato et al., 2014). In our study we found that the IC_50_ for decreasing off-contractions is around 100 μg/mL, which corresponds to one capsule (186 mg) diluted in 1.8 L. This value is in the same range as the IC_50_ (around 50 µM) observed for calcium current reduction measured with patch-clamp (Hills and Aaronson, 1991). A similar IC_50_ value can be estimated for the reduction of myogenic contractions and cch-induced contractions. Slightly higher concentrations are needed to reduce neuron-mediated excitatory responses in both muscle layers. The effect of peppermint oil on contractility is similar to menthol (Amato et al., 2014), suggesting that menthol, which is the major constituent of peppermint oil, is important in mediating its actions. Interestingly, this study provides for the first time data from the longitudinal layer, since previous published works were mainly focused on the circular muscle (Amato et al., 2014; Krueger et al., 2020).

Since both HBB and peppermint oil reduced spontaneous, cch- and EFS-induced contractions, we performed an isobolographic study to test for potential synergism. Consistent with a synergistic effect between HBB and peppermint oil, the interaction index was less than 1 for all measured parameters and muscle layers. It is possible that the two mechanisms of action, i.e., antimuscarinic and L-type calcium channel blocker for HBB and peppermint oil respectively, are the basis for this synergism. These results suggest that the spasmolytic effect of these drugs in combination may be more potent than separately. This result could be relevant for patients and future prescription drugs. In conclusion, this study shows that drotaverine and HBB have a complementary effect based on their mechanisms of action on human colon smooth muscle. While HBB is mainly an antimuscarinic drug that reduces the effect of excitatory pathway, drotaverine enhances the relaxation attributed to cAMP, acting as a PDE4 inhibitor. Paracetamol at therapeutic concentrations did not modify muscle contractility. However, at supratherapeutic concentrations, a slight decrease in contractility probably associated to L-type calcium channel inhibition could be observed. The association of paracetamol and HBB was not different from HBB alone, suggesting no complementary effect on motility at therapeutic doses. This indicates that the long-established association of these two drugs provides combined analgesic effects by paracetamol and spasmolytic effects by HBB. In contrast, peppermint oil reduces spontaneous contractions, cch responses, and neural-mediated excitatory responses alone probably via an L-type calcium channel inhibitory effect and, when combined with HBB, shows a synergistic effect. This might have important clinical implications, as combined treatment may offer treatment benefits not seen with monotherapy.

## Data Availability

The raw data on which the conclusions are based may be available upon request.

## Supplementary material

SUPPLEMENTARY FIGURE S1 The effect of paracetamol and peppermint oil (PPO) on spontaneous phasic contractions. Frequency, amplitude, and tone of spontaneous phasic contractions in the presence of paracetamol **(A)** and PPO **(B,C)**. Data were normalized (i.e., 100%) to the basal. Dunnett’s post-hoc test was done after ANOVA. **P* < 0.05, ***P* < 0.01, ^****^ *P* < 0.0001 compared with basal conditions. Data were expressed as mean ± SEM.

SUPPLEMENTARY TABLE S1 Patient characteristics.

SUPPLEMENTARY TABLE S2 Composition of peppermint oil.

## References

[B1] Al-Shboul O. Mahavadi S. Sriwai W. Grider J. R. Murthy K. S. (2013). Differential expression of multidrug resistance protein 5 and phosphodiesterase 5 and regulation of cGMP levels in phasic and tonic smooth muscle. Am. J. Physiol. Gastrointest. Liver Physiol. 305 (4), G314–G324. 10.1152/ajpgi.00457.2012 23764893 PMC3891211

[B2] Amato A. Liotta R. Mule F. (2014). Effects of menthol on circular smooth muscle of human colon: analysis of the mechanism of action. Eur. J. Pharmacol. 740, 295–301. 10.1016/j.ejphar.2014.07.018 25046841

[B3] Auli M. Martinez E. Gallego D. Opazo A. Espin F. Marti-Gallostra M. (2008). Effects of excitatory and inhibitory neurotransmission on motor patterns of human sigmoid colon *in vitro* . Br. J. Pharmacol. 155 (7), 1043–1055. 10.1038/bjp.2008.332 18846038 PMC2597251

[B4] Bannwarth B. Netter P. Lapicque F. Gillet P. Pere P. Boccard E. (1992). Plasma and cerebrospinal fluid concentrations of paracetamol after a single intravenous dose of propacetamol. Br. J. Clin. Pharmacol. 34 (1), 79–81. 10.1111/j.1365-2125.1992.tb04112.x 1633071 PMC1381380

[B5] Barnette M. S. Manning C. D. Price W. J. Barone F. C. (1993). Initial biochemical and functional characterization of cyclic nucleotide phosphodiesterase isozymes in canine colonic smooth muscle. J. Pharmacol. Exp. Ther. 264 (2), 801–812. 7679736

[B6] Barone F. C. Barton M. E. White R. F. Legos J. J. Kikkawa H. Shimamura M. (2008). Inhibition of phosphodiesterase type 4 decreases stress-induced defecation in rats and mice. Pharmacology 81 (1), 11–17. 10.1159/000107662 17726343

[B7] Blair N. T. Caceres A. I. Carvacho I. Chaudhuri D. Clapham D. E. De Clercq K. (2023). Transient receptor potential channels (TRP) in GtoPdb v.2023.3. IUPHAR/BPS Guide Pharmacol. CITE. 2023 (3). 10.2218/gtopdb/F78/2023.3

[B8] Bolaji O. O. Onyeji C. O. Ogundaini A. O. Olugbade T. A. Ogunbona F. A. (1996). Pharmacokinetics and bioavailability of drotaverine in humans. Eur. J. Drug Metab. Pharmacokinet. 21 (3), 217–221. 10.1007/BF03189716 8980918

[B9] Broad J. Kung V. W. S. Palmer A. Elahi S. Karami A. Darreh-Shori T. (2019). Changes in neuromuscular structure and functions of human colon during ageing are region-dependent. Gut 68 (7), 1210–1223. 10.1136/gutjnl-2018-316279 30228216 PMC6594449

[B10] Carbone S. E. Dinning P. G. Costa M. Spencer N. J. Brookes S. J. Wattchow D. A. (2013). Ascending excitatory neural pathways modulate slow phasic myogenic contractions in the isolated human colon. Neurogastroenterol. Motil. 25 (8), 670–676. 10.1111/nmo.12129 23634776

[B11] Chen L. Ilham S. J. Feng B. (2017). Pharmacological approach for managing pain in irritable bowel syndrome: a review article. Anesth. Pain Med. 7 (2), e42747. 10.5812/aapm.42747 28824858 PMC5556397

[B12] Chen Y. Y. Yu M. F. Zhao X. X. Shen J. Peng Y. B. Zhao P. (2020). Paracetamol inhibits Ca(2+) permeant ion channels and Ca(2+) sensitization resulting in relaxation of precontracted airway smooth muscle. J. Pharmacol. Sci. 142 (2), 60–68. 10.1016/j.jphs.2019.07.007 31843508

[B13] Chou T. C. (2006). Theoretical basis, experimental design, and computerized simulation of synergism and antagonism in drug combination studies. Pharmacol. Rev. 58 (3), 621–681. 10.1124/pr.58.3.10 16968952

[B14] Correia M. C. Santos E. S. A. Neves B. J. Rocha M. L. (2022). Acetaminophen treatment evokes anticontractile effects in rat aorta by blocking L-type calcium channels. Pharmacol. Rep. 74 (3), 493–502. 10.1007/s43440-022-00367-y 35438421

[B15] Corsetti M. Costa M. Bassotti G. Bharucha A. E. Borrelli O. Dinning P. (2019). First translational consensus on terminology and definitions of colonic motility in animals and humans studied by manometric and other techniques. Nat. Rev. Gastroenterol. Hepatol. 16 (9), 559–579. 10.1038/s41575-019-0167-1 31296967 PMC7136172

[B16] Corsetti M. Forestier S. Jimenez M. (2023). Hyoscine butylbromide mode of action on bowel motility: from pharmacology to clinical practice. Neurogastroenterol. Motil. 35 (4), e14451. 10.1111/nmo.14451 35972266

[B17] Couso E. Hidalgo A. Cantabrana B. (1996). Spasmolytic effect of paracetamol on rat uterine smooth-muscle contraction. Pharm. Pharmacol. Commun. 2 (3), 145–147. 10.1111/j.2042-7158.1996.tb00580.x

[B18] Donnerer J. Liebmann I. (2017). Effects of allyl isothiocyanate, acetaminophen, and dipyrone in the Guinea-pig ileum. Pharmacology 99 (1-2), 79–83. 10.1159/000452164 27756064

[B19] Forrest J. A. Clements J. A. Prescott L. F. (1982). Clinical pharmacokinetics of paracetamol. Clin. Pharmacokinet. 7 (2), 93–107. 10.2165/00003088-198207020-00001 7039926

[B20] Gallego D. Gil V. Aleu J. Auli M. Clave P. Jimenez M. (2008). Purinergic and nitrergic junction potential in the human colon. Am. J. Physiol. Gastrointest. Liver Physiol. 295 (3), G522–G533. 10.1152/ajpgi.00510.2007 18599588

[B21] Gallego D. Hernandez P. Clave P. Jimenez M. (2006). P2Y1 receptors mediate inhibitory purinergic neuromuscular transmission in the human colon. Am. J. Physiol. Gastrointest. Liver Physiol. 291 (4), G584–G594. 10.1152/ajpgi.00474.2005 16751171

[B22] Graham G. G. Davies M. J. Day R. O. Mohamudally A. Scott K. F. (2013). The modern pharmacology of paracetamol: therapeutic actions, mechanism of action, metabolism, toxicity and recent pharmacological findings. Inflammopharmacology 21 (3), 201–232. 10.1007/s10787-013-0172-x 23719833

[B23] Hartmann G. Bidlingmaier C. Siegmund B. Albrich S. Schulze J. Tschoep K. (2000). Specific type IV phosphodiesterase inhibitor rolipram mitigates experimental colitis in mice. J. Pharmacol. Exp. Ther. 292 (1), 22–30. 10604928

[B24] Herbert M. K. Weis R. Holzer P. Roewer N. (2005). Peristalsis in the Guinea pig small intestine *in vitro* is impaired by acetaminophen but not aspirin and dipyrone. Anesth. Analg. 100 (1), 120–127. 10.1213/01.ANE.0000139352.54676.18 15616065

[B25] Hills J. M. Aaronson P. I. (1991). The mechanism of action of peppermint oil on gastrointestinal smooth muscle. An analysis using patch clamp electrophysiology and isolated tissue pharmacology in rabbit and Guinea pig. Gastroenterology 101 (1), 55–65. 10.1016/0016-5085(91)90459-x 1646142

[B26] Jozwiak-Bebenista M. Nowak J. Z. (2014). Paracetamol: mechanism of action, applications and safety concern. Acta Pol. Pharm. 71 (1), 11–23. 24779190

[B27] Krueger D. Schauffele S. Zeller F. Demir I. E. Theisen J. Michel K. (2020). Peppermint and caraway oils have muscle inhibitory and pro-secretory activity in the human intestine *in vitro* . Neurogastroenterol. Motil. 32 (2), e13748. 10.1111/nmo.13748 31612595

[B28] Milligan T. P. Morris H. C. Hammond P. M. Price C. P. (1994). Studies on paracetamol binding to serum proteins. Ann. Clin. Biochem. 31 (Pt 5), 492–496. 10.1177/000456329403100512 7832576

[B29] Mousavi T. Sharifnia M. Nikfar S. Abdollahi M. (2023). Pharmacotherapy for gastric and intestinal cramping pain: current and emerging therapies. Expert Opin. Pharmacother. 24 (18), 2021–2033. 10.1080/14656566.2023.2265830 37788098

[B30] Muller-Lissner S. Andresen V. Corsetti M. Bustos Fernandez L. Forestier S. Pace F. (2022). Functional abdominal cramping pain: expert practical guidance. J. Clin. Gastroenterol. 56 (10), 844–852. 10.1097/MCG.0000000000001764 36149666 PMC9553264

[B31] NLM (2023). National center for biotechnology information. PubChem compound summary for CID 1712095, drotaverine.

[B32] Patai Z. Guttman A. Mikus E. G. (2016). Potential L-type voltage-operated calcium channel blocking effect of drotaverine on functional models. J. Pharmacol. Exp. Ther. 359 (3), 442–451. 10.1124/jpet.116.237271 27738091

[B33] Patai Z. Guttman A. Mikus E. G. (2018). Assessment of the airway smooth muscle relaxant effect of drotaverine. Pharmacology 101 (3-4), 163–169. 10.1159/000485921 29301136

[B34] Pauwelyn V. Ceelen W. Lefebvre R. A. (2018). Synergy between 5-HT(4) receptor stimulation and phosphodiesterase 4 inhibition in facilitating acetylcholine release in human large intestinal circular muscle. Neurogastroenterol. Motil. 30 (2). 10.1111/nmo.13162 28799255

[B35] Peier A. M. Moqrich A. Hergarden A. C. Reeve A. J. Andersson D. A. Story G. M. (2002). A TRP channel that senses cold stimuli and menthol. Cell 108 (5), 705–715. 10.1016/s0092-8674(02)00652-9 11893340

[B36] Priem E. K. De Maeyer J. H. Lefebvre R. A. (2013). Influence of phosphodiesterases on basal and 5-HT₄ receptor facilitated cholinergic contractility in pig descending colon. Eur. J. Pharmacol. 705 (1-3), 156–163. 10.1016/j.ejphar.2013.02.011 23454061

[B37] Przybyla G. W. Szychowski K. A. Gminski J. (2020). Paracetamol - an old drug with new mechanisms of action. Clin. Exp. Pharmacol. Physiol. 48, 3–19. 10.1111/1440-1681.13392 32767405

[B38] Rae M. G. Fleming N. McGregor D. B. Sanders K. M. Keef K. D. (1998). Control of motility patterns in the human colonic circular muscle layer by pacemaker activity. J. Physiol. 510 (Pt 1), 309–320. 10.1111/j.1469-7793.1998.309bz.x 9625887 PMC2231034

[B39] Rettenbacher M. Reubi J. C. (2001). Localization and characterization of neuropeptide receptors in human colon. Naunyn Schmiedeb. Arch. Pharmacol. 364 (4), 291–304. 10.1007/s002100100454 11683516

[B40] Tanner T. Aspley S. Munn A. Thomas T. (2010). The pharmacokinetic profile of a novel fixed-dose combination tablet of ibuprofen and paracetamol. BMC Clin. Pharmacol. 10, 10. 10.1186/1472-6904-10-10 20602760 PMC2906415

[B41] Tomoskozi Z. Finance O. Aranyi P. (2002). Drotaverine interacts with the L-type Ca(2+) channel in pregnant rat uterine membranes. Eur. J. Pharmacol. 449 (1-2), 55–60. 10.1016/s0014-2999(02)01993-3 12163106

[B42] Traserra S. Alcala-Gonzalez L. Barber C. Landolfi S. Malagelada C. Corsetti M. (2024). New insights into the characterization of the mechanism of action of hyoscine butylbromide in the human colon *ex vivo* . Eur. J. Pharmacol. 972, 176550. 10.1016/j.ejphar.2024.176550 38570081

[B43] Vane J. R. Botting R. M. (1998). Anti-inflammatory drugs and their mechanism of action. Inflamm. Res. 47 (Suppl. 2), S78–S87. 10.1007/s000110050284 9831328

[B44] Videla S. Vilaseca J. Medina C. Mourelle M. Guarner F. Salas A. (2006). Selective inhibition of phosphodiesterase-4 ameliorates chronic colitis and prevents intestinal fibrosis. J. Pharmacol. Exp. Ther. 316 (2), 940–945. 10.1124/jpet.105.090837 16254133

[B45] Yuan F. Ren H. Tan W. Wang Y. Luo H. (2022). Effect of phosphodiesterase-4 inhibitor rolipram on colonic hypermotility in water avoidance stress rat model. Neurogastroenterol. Motil. 34 (7), e14317. 10.1111/nmo.14317 35037375 PMC9286810

