# Supplementary material for: Evaluation of the mechanism of action of paracetamol, drotaverine, and peppermint oil and their effects in combination with hyoscine butylbromide on colonic motility: human ex-vivo study
Source: Front Pharmacol. 2024 Jul 10;15:1384070. doi: 10.3389/fphar.2024.1384070 (PMC11266310; doi:10.3389/fphar.2024.1384070)
Supplement: Supplementary file 1 [file Table1.docx]

**Supplementary Table 1. Patient characteristics**

|  |  | **Right colon** |  | **Left colon** |
| --- | --- | --- | --- | --- |
| Number of patients |  | 34 |  | 34 |
| Gender |  | 12 women/22 men |  | 14 women/20 men |
| Age range (mean ± SD) |  | 38–93 years (74.3 years ± 11.59) |  | 35–90 years (68.3 years ± 15.16) |
