# Supplementary figures and images for: Evaluation of the mechanism of action of paracetamol, drotaverine, and peppermint oil and their effects in combination with hyoscine butylbromide on colonic motility: human ex-vivo study
Source: Front Pharmacol. 2024 Jul 10;15:1384070. doi: 10.3389/fphar.2024.1384070 (PMC11266310; doi:10.3389/fphar.2024.1384070)

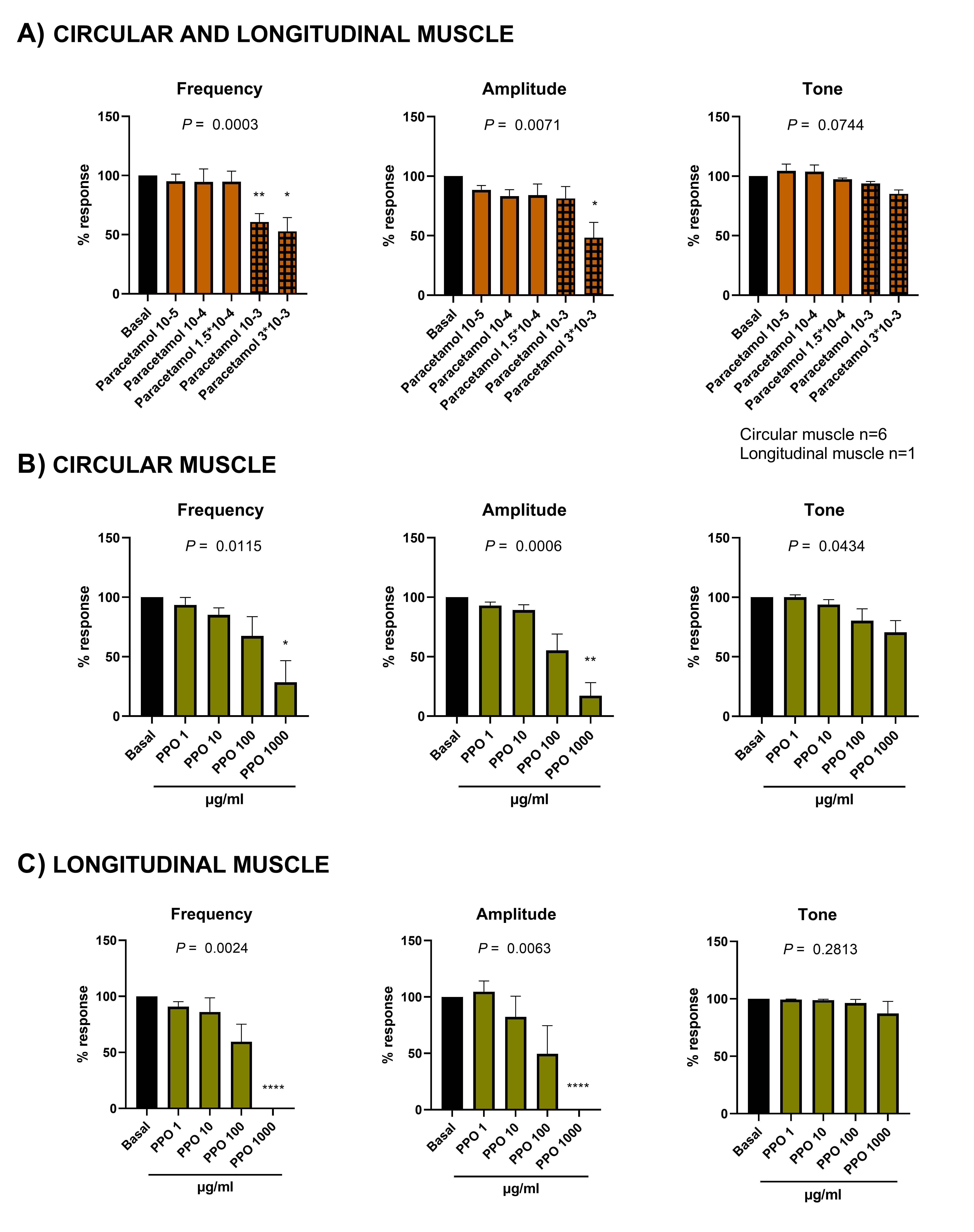

Supplement: Supplementary file 2 [file Image1.tif]
