# Supplementary material for: Evaluation of the mechanism of action of paracetamol, drotaverine, and peppermint oil and their effects in combination with hyoscine butylbromide on colonic motility: human ex-vivo study
Source: Front Pharmacol. 2024 Jul 10;15:1384070. doi: 10.3389/fphar.2024.1384070 (PMC11266310; doi:10.3389/fphar.2024.1384070)
Supplement: Supplementary file 3 [file Table2.docx]

**Supplementary Table 2. Composition of peppermint oil**

| Characteristic | Value | Unit | Lower Limit | Upper  Limit |
| --- | --- | --- | --- | --- |
| Appearance (sensorical) | Corresponds | clear Liquid |  |  |
| Colour (sensorical) | Corresponds | colourless or pale yellow to pale greenish-yellow |  |  |
| Odour (sensorical) | Corresponds | characteristic |  |  |
| Relative density (20/20) | 0.901 |  | 0.900 | 0.916 |
| Refractive index (at 20°C) | 1.459 |  | 1.457 | 1.467 |
| Optical rotation (at 20°C) | -27.3 |  | -30.0 | -10.0 |
| Solubility in Ethanol 96% | Corresponds |  |  |  |
| Acid Value (with 5g oil) | 0.1 |  |  | 1.4 |
| Fatty resinified volatile oils | Corresponds |  |  |  |
| Water | Corresponds |  |  |  |
| Chromatographic Profile | corresponds |  |  |  |
| Carvone | 0.0 | A% |  | 1.0 |
| 1.8-Cineole | 6.1 | A% | 3.5 | 8.0 |
| Isomenthone | 3.7 | A% | 1.5 | 10.0 |
| Isopulegol | 0.1 | A% |  | 0.2 |
| Limonene | 2.6 | A% | 1.0 | 3.5 |
| Menthofurane | 1.5 | A% | 1.0 | 3.0 |
| Menthol | 43.0 | A% | 30.0 | 55.0 |
| Menthone | 24.7 | A% | 14.0 | 32.0 |
| Menthylacetate | 5.7 | A% | 2.8 | 10.0 |
| Pulegone | 0.8 | A% |  | 2.4 |
| Pulegone + Menthoforane | 2 | A% |  | 3 |
| Ratio of 1.8- Cincol content to Limo | 2.3 |  | 2.0 |  |
| Mint oil B.: Isopulegol | 0.1 | A% |  | 0.2 |
| Special impurities: Tests on heavy metals, pesticides, aflatoxins and microbiology are carried out on the oil used for vacuum distillation (educt). | | | | |
